# Maternal and Indoor Smoking Are Associated with Increased Risk of Acute Respiratory Infection in Under-Five Children: A Meta-Analysis of National Survey Data from Eight Asia-Pacific Countries

**DOI:** 10.3390/healthcare14193221

**Published:** 2026-09-30

**Authors:** Daniel Bogale Odo, Yonatan Dinku, Uday Narayan Yadav, Desalegn Markos Shifti, Zelalem Mengesha, Giridhara Rathnaiah Babu, Bhim Prasad Sapkota, Raglan Maddox

**Affiliations:** 1 National Centre for Aboriginal and Torres Strait Islander Wellbeing Research, National Centre for Epidemiology and Population Health, The Australian National University, Canberra, ACT 2601, Australia; raglan.maddox@anu.edu.au; 2 Centre for Indigenous Policy Research, The Australian National University, Canberra, ACT 2601, Australia; yonatan.dinku@anu.edu.au; 3 National Centre for Epidemiology and Population Health, The Australian National University, Canberra, ACT 2601, Australia; uday.yadav@anu.edu.au; 4 Center for Research Policy and Implementation, Biratnagar 56613, Nepal; 5 International Centre for Future Health Systems, University of New South Wales, Sydney, NSW 2033, Australia; 6 Child Health Research Centre, The University of Queensland, Brisbane, QLD 4101, Australia; d.shifti@uq.edu.au; 7 Health Research Institute, University of Canberra, Canberra, ACT 2617, Australia; zelalem.mengesha@canberra.edu.au; 8 Department of Population Medicine, College of Medicine, QU Health, Qatar University, Doha 2713, Qatar; epigiridhar@gmail.com; 9 Ministry of Health and Population, Government of Nepal, Kathmandu 44600, Nepal; bhim.sapkota@nepal.gov.np; 10 Bagumani (Modewa) Clan, Papua New Guinea

**Keywords:** tobacco use, maternal smoking, indoor smoking, respiratory infections, child health

## Abstract

**Background**: Early exposure to tobacco smoke can harm children’s developing respiratory systems, increasing the risk of acute and long-term health consequences. This study examined the association of maternal and indoor smoking with acute respiratory infection (ARI) in under-five children living in eight Asia-Pacific countries. **Methods**: We analysed Demographic and Health Survey data collected between 2015 and 2022. A logistic regression model was fitted separately to each country’s data to obtain the odds of ARI in children for maternal tobacco use, daily indoor smoking, and occasional indoor smoking. Then, a meta-analysis using the Der Simonian and Laird method was used to obtain a pooled odds ratio (pOR) of ARI across the included countries. **Results**: We included a weighted sample of 302,841 children, among whom the prevalence of ARI was 4.12% (95% CI: 4.05, 4.19). The pooled prevalence of maternal tobacco use and daily indoor smoking was 3.45% (95% CI: 3.38, 3.51) and 29.61% (95% CI: 29.45, 29.77), respectively. In random-effects meta-analyses, exposure to daily indoor smoking (pOR = 1.13; 95% CI: 1.02, 1.25) and occasional indoor smoking (pOR = 1.23; 95% CI: 1.13, 1.35) were both associated with increased odds of ARI, as was maternal tobacco use (pOR = 1.67; 95% CI: 1.21, 2.31). **Conclusions**: Secondhand smoke exposure was associated with higher odds of ARI among under-five children, underscoring the need for smoke-free home policies across Asia-Pacific countries, particularly as the tobacco industry expands marketing and lobbying against such policies in low-income settings.

## 1. Introduction

Acute respiratory infection (ARI) remains one of the leading causes of morbidity and mortality among under-five children worldwide [1], with the greatest burden concentrated in low- and middle-income countries (LMICs) [2]. Substantial progress has been made in reducing childhood ARI mortality, largely driven by vaccination, improved nutrition, and access to care [3]. However, environmental risk factors such as tobacco smoke exposure and household air pollution continue to undermine this progress [4,5], particularly in LMICs where secondhand smoke (SHS) exposure is among the highest compared to high-income countries [6].

Tobacco kills more than 7 million people worldwide each year, including 1.3 million who die from SHS exposure [7]. Exposure to tobacco smoke, whether before or after birth, is linked to a wide range of harms in children’s health [8,9]. Prenatal tobacco exposure restricts foetal growth and raises the risk of low birthweight, stillbirth, neonatal death, and birth defects [10]. It also impairs lung development and contributes to neurodevelopmental problems [11,12]. Children exposed in utero also face higher risks of overweight and obesity, elevated blood pressure, vision problems, and reproductive health issues later in life [8]. After birth, continued exposure through breastfeeding or secondhand or/and thirdhand smoke in the home compounds these risks [13,14], such as pneumonia [15], asthma [16], and sudden infant death syndrome [11,17]. Additionally, children’s respiratory health consequences from SHS exposure carry significant social and economic burdens. Together, this evidence shows that tobacco smoke exposure during critical developmental periods affects nearly every body system, with consequences that often persist into adulthood.

Evidence suggests a dose–response relationship between tobacco smoke exposure and respiratory outcomes in children, with the frequency and intensity of indoor smoking likely shaping the degree of risk [17,18]. However, much of the existing literature treats SHS exposure as a binary condition, without distinguishing between children routinely exposed through daily indoor smoking and those exposed only occasionally. Disentangling the independent and relative contributions of maternal smoking, daily indoor smoking, and occasional indoor smoking to respiratory infection risk in under-five children is therefore essential to inform more targeted public health interventions. Much of the available evidence has also been derived either from single-country [19] or older data [20], limiting its generalisability to the diverse socioeconomic and cultural contexts.

A central driver of this ongoing burden is the tobacco industry’s deliberate strategic shift toward LMICs [21,22]. This shift has been accompanied by sustained industry lobbying against smoke-free legislation, including delaying the World Health Organization’s Framework Convention on Tobacco Control (WHO FCTC) implementation, litigation, trade disputes, and influencing policymakers to weaken proposed initiatives [23,24]. The consequence is that many households lack the legislative and social infrastructure to protect children from indoor tobacco smoke exposure, even as awareness of its harms grows [25]. Because young children spend most of their time at home, they face disproportionate SHS exposure in settings least reached by tobacco control, underscoring the need for household-level evidence to inform policy.

To address these gaps, we conducted a meta-analysis of the most recent Demographic and Health Survey (DHS) data from eight Asia-Pacific countries. The Asia-Pacific region has some of the highest rates of pneumonia illness and death in the world, with countries such as India, Indonesia, and Pakistan ranking among the highest-burden nations worldwide [26,27]. Compounding this burden, the region also exhibits elevated tobacco consumption rates [28]. Despite this substantial burden, existing research has largely been confined to single-country analyses [19] and relies on data that may no longer reflect current disease patterns [20]. We examined the association between three distinct exposure scenarios—maternal tobacco use, daily indoor smoking, and occasional indoor smoking—and the odds of ARI among under-five children. In this study, ‘Asia-Pacific’ refers to the specific countries included in the analysis rather than the region as a whole. By comparing risks across exposure patterns in a large, multi-country dataset, this study provides robust evidence to inform smoke-free home policies at least in the included countries.

## 2. Methods

### 2.1. Data and Data Collection Procedures

**Figure 1:**
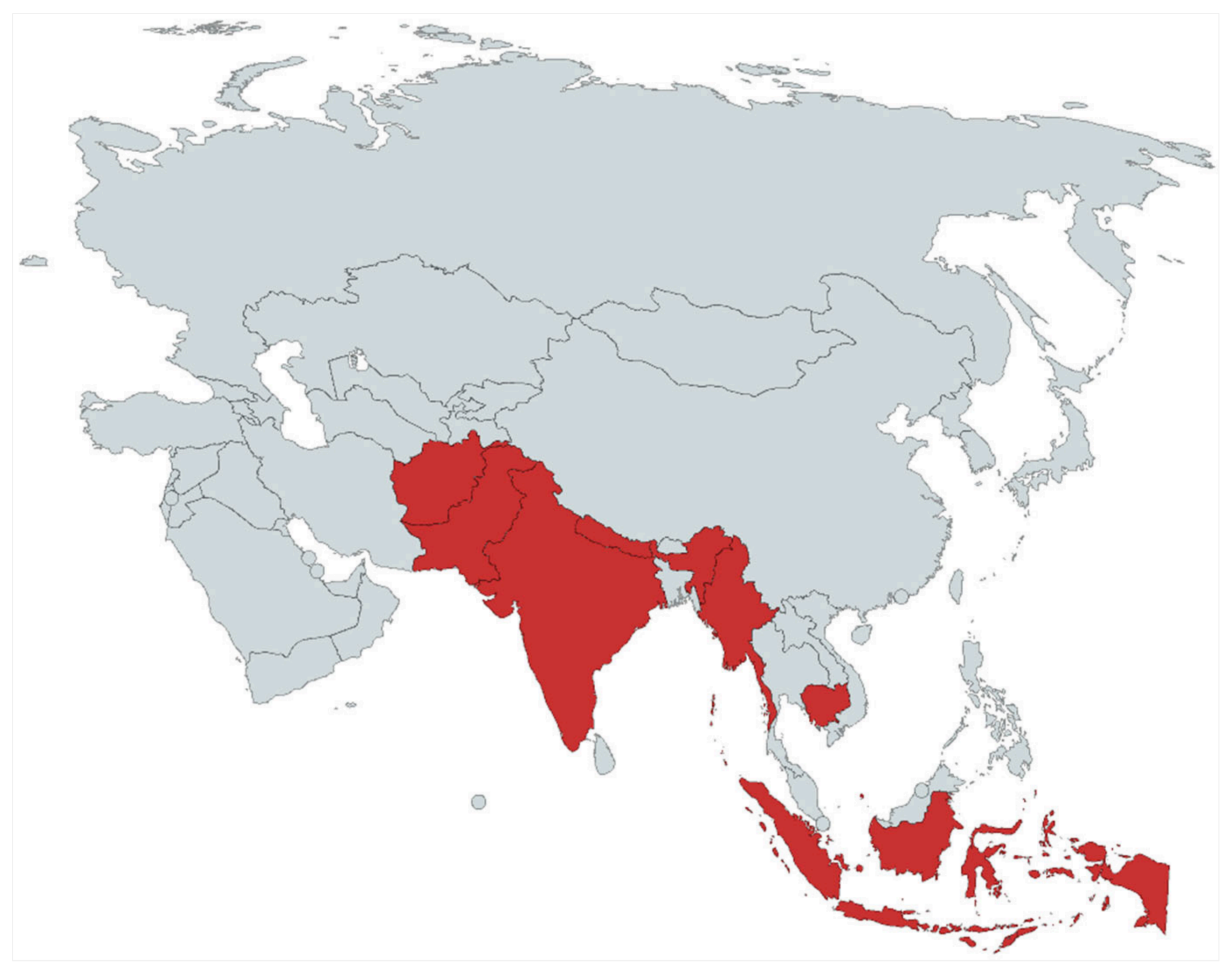
Countries included in the study (shaded in red). Map created at www.mapchart.net, extent trimmed for visualisation.

This study used DHS data from eight countries in the Asia-Pacific region: Afghanistan, Cambodia, Indonesia, India, Myanmar, Nepal, Pakistan, and Timor-Leste (Figure 1). DHS generates representative health and health-related indicators at national and sub-national levels. Briefly, DHS uses a two-stage stratified cluster sampling technique in which clusters are selected from a stratum with probability proportional to the size of enumeration areas (EAs), obtained commonly from the nearest population census. These EAs correspond roughly with a village or a neighbourhood. Then, households are randomly selected in each cluster, and women aged 15–49 years are interviewed from those households. DHS has multiple data files, and for this analysis, we used kids’ records (KR) and household records (HR).

### 2.2. Inclusion Criteria

The included countries were selected from the Asia-Pacific region based on the availability of variables of interest and the completeness of the DHS data. Countries were excluded if they lacked data on maternal or household smoking, had inadequate sample for inferential analysis, or did not collect data on ARI. Details of the inclusion and exclusion process are provided in Supplementary Materials (Figure S1; Table S1).

### 2.3. Dependent Variable

The outcome of interest was ARI in children aged <5 years, derived from a set of specific questions. In an effort to reduce pneumonia mortality in children, the WHO developed a case management approach for low-resource settings, which classified ARI (pneumonia) in children as a cough accompanied by rapid breaths alone. This definition was modified in the revised version by the addition of chest indrawing; cough accompanied by short, rapid breathing, which was chest-related; and/or by difficult breathing, which was chest-related as indicative of simple pneumonia [29]. In the DHS, women were asked whether their children suffered/experienced fast or rapid breathing or not and whether the difficulty noticed in breathing was due to blocked nose and chest congestion in the last two weeks before the interview. The child’s ARI status is then computed based on responses to these questions. The variables used to calculate the number of children with ARI were h31b and h31c. See Table S2 for the complete algorithm and STATA code used.

### 2.4. Exposure Variables

Maternal tobacco use and frequency of smoking inside the house (indoor smoking) were the exposure variables. These exposure variables were computed based on the assumption that all forms of commercial tobacco products are harmful and that there is no safe level of exposure to commercial tobacco smoke [7]. Maternal tobacco use in this study refers specifically to any form of tobacco products (cigarettes and other tobacco products) captured in the DHS questionnaire. This was measured in the survey as whether a mother used any form of tobacco products such as cigarettes, shisha, cigars, and a pipe full of tobacco and recorded as yes or no for every tobacco product. This was then summarised into a single question (yes/no) about whether a mother used any form of tobacco products. The second exposure variable (indoor smoking) was measured as the frequency of household members smoking inside the house, which was recorded as never, daily, weekly, monthly, and less than once monthly. Three categories were formed from this variable after merging the weekly, monthly, and less than once monthly categories into one: never, daily, and occasionally. Occasional smoking was defined as smoking on a less than daily basis. We followed the Guide to DHS Statistics, national tobacco surveys, and previous studies that used similar categories, which also allowed a consistent definition across countries (see Table S2).

### 2.5. Covariates

Covariates were selected based on the available evidence [19,30] on the association between tobacco use and ARI in children. These include place of residence, type of cooking fuel (as a proxy for household air pollution), hand hygiene (measured as having a handwashing facility), household wealth index, number of household members (as a proxy for crowding index), maternal education and age, and child sex and age. A detailed explanation of these variables is in the supplement (Table S2). Additionally, maternal tobacco use and indoor smoking, the two main exposure variables, were each adjusted for the other in the analysis.

### 2.6. Statistical Analysis

We used descriptive statistics to characterise the study population and bivariate cross-tabulations to calculate the prevalence of ARI based on the children’s sociodemographic characteristics. Observations with missing values on any variable included were excluded from the analysis. In each dataset, cluster number and country code were used to form a new cluster variable, and country code, type of place of residence, and state were used to form a new stratum variable. Then, we used STATA’s *svyset* command to account for the resulting weighting, clustering, and stratification, specifying the DHS-supplied sampling weight, the new cluster variable, and strata. We then used *svy* to calculate weighted descriptive statistics both for country-specific and pooled data and logistic regression to estimate odds ratios (ORs) for the association between maternal tobacco use and SHS exposure and childhood ARI. The included countries may have varying cultural, economic, and health characteristics, so the specific context of each country needs to be considered when examining the relationship between commercial tobacco use/SHS exposure and child health outcomes. As such, a separate logistic regression model was fitted for each country so that independent ORs of ARI were produced for each country.

Then, the random-effects meta-analysis was performed using the ‘metan’ command following the approach described by Der Simonian and Laird [31] to obtain a pooled odds ratio (pOR) for each exposure variable across the countries included. We pooled country-specific estimates to synthesize evidence across surveys with comparable DHS methodology, not to represent the Asia-Pacific region as a whole. This technique has been used in previous studies [32]. Between-study (i.e., country-level estimate) heterogeneity was quantified using the I^2^ statistic and *p*-value. We calculated the 95% prediction interval for each pooled effect estimate to assess the range within which the true effect size would fall in 95% of comparable future studies. Additionally, a fixed-effects model was used as a sensitivity analysis by giving more weight for larger studies than smaller studies [33], and a random-effects model may have issues achieving optimal error estimation [34].

All analyses were conducted using STATA software (version STATA/SE 18; College Station, TX, USA: StataCorp LLC.).

## 3. Results

Overall, 12,492 of the 302,841 weighted sample of children pooled across the eight countries had ARI, giving a prevalence of 4.12% (95% CI: 4.05, 4.19). Country-specific prevalence varied widely, from 1.26% in Cambodia to 13.81% in Pakistan. The pooled and weighted prevalence of maternal tobacco use was 3.45% (95% CI: 3.38, 3.51), ranging from 1.88% in Cambodia to 6.96% in Pakistan (Table 1).

**Figure 2:**
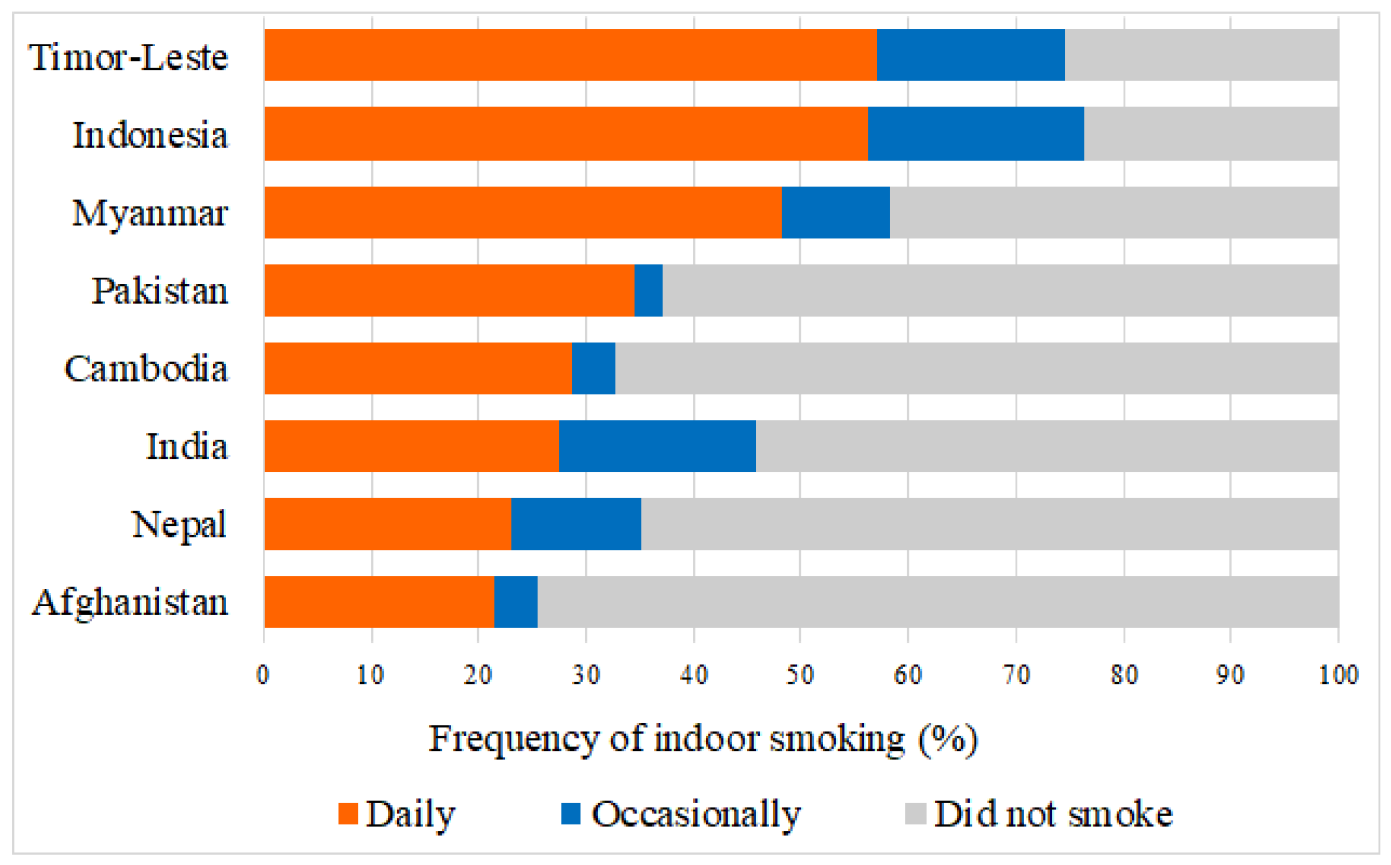
Weighted frequency of indoor smoking by Asia-Pacific countries, DHS data (2015–2022). Results are sorted by daily indoor smoking.

The pooled and weighted prevalence of daily smoking inside the house was 29.61% (95% CI: 29.45, 29.77), ranging from 21.50% in Afghanistan to 56.96% in Timor-Leste. The pooled prevalence of occasional smoking inside the house was 15.79% (95% CI: 15.66, 15.92), ranging from 2.59% in Pakistan to 19.95% in Indonesia (Figure 2 and Table S3).

In Timor-Leste, the prevalence of ARI among children of mothers who used tobacco was roughly seven percentage points higher than among children of mothers who did not (8.67% vs. 1.62%). A similarly sizeable gap between children of mothers who did and did not use tobacco was observed in India (4.09% vs. 2.75%), Pakistan (18.9% vs. 13.62%), Cambodia (5.25% vs. 1.18%), and Afghanistan (16.71% vs. 12.42%). Children in households with daily or occasional indoor smoking generally had slightly greater ARI prevalence than those with no indoor smoking. Further details on this and the relationship between ARI and other individual, household, and contextual factors are provided in the Supplementary File (Table S4).

### 3.1. Association Between Maternal Tobacco Use and ARI in Children

**Figure 3:**
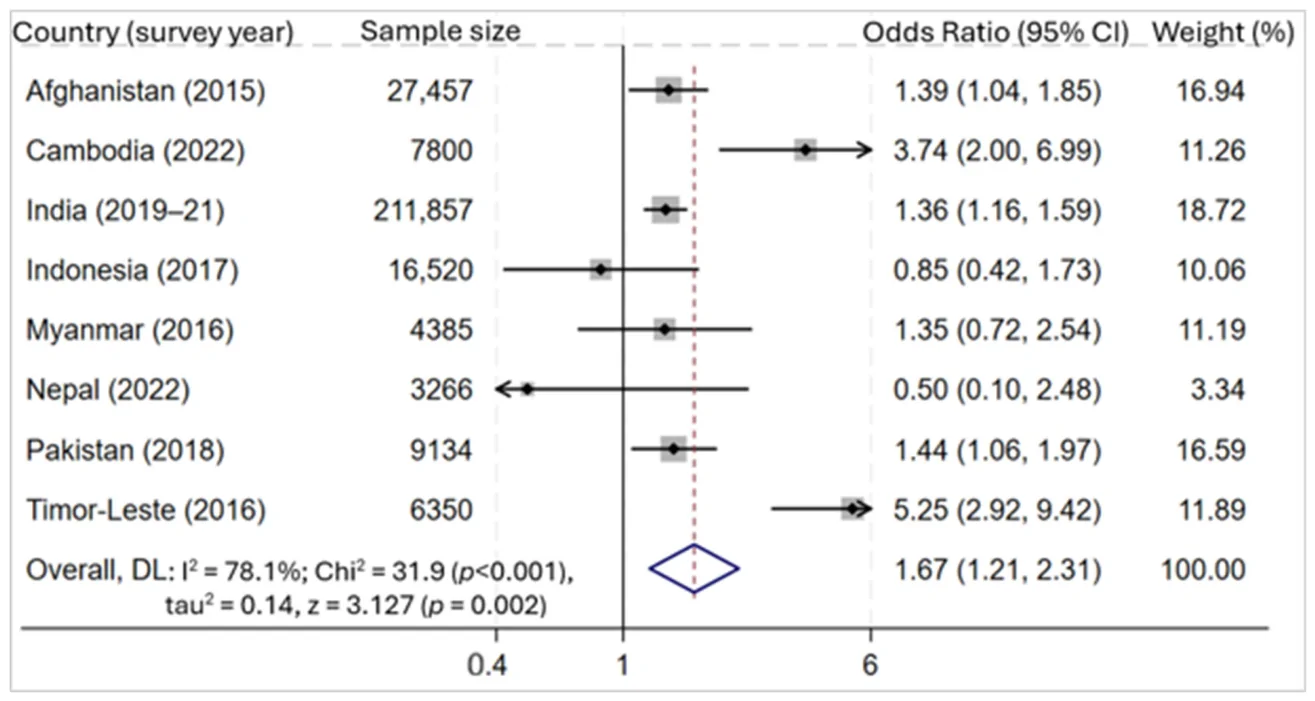
Odds ratio of child ARI associated with maternal tobacco use in the Asia-Pacific region. The diamond symbols and size of the shaded grey boxes indicate effect size and the contribution of country-specific estimates in the meta-analysis, respectively, and horizontal lines indicate 95% CIs. The centre of the blue diamond denotes the pooled OR, and the corners indicate the 95% confidence intervals. The sample size shown in the figure is unweighted.

Tobacco use by mothers was associated with higher odds of ARI in children aged <5 years in five of the eight included countries: estimates ranged from OR = 5.25 (95% CI: 2.92, 9.42) in Timor-Leste to OR = 1.36 (95% CI: 1.16, 1.59) in India (Table S5). The pooled estimate showed a higher odds of ARI (pOR = 1.67, 95% CI: 1.21, 2.31) in children whose mothers were tobacco users. A substantial difference between country-specific estimates was observed (I2: 78.1%), depicting pronounced heterogeneity in the odds of ARI in children associated with maternal tobacco use between countries (Figure 3). Given the observed substantial heterogeneity, we calculated the 95% prediction interval (0.62, 4.51), which suggests that the true effect in a new country estimate (study) could range from modestly protective to considerably harmful association.

### 3.2. Association Between Frequency of Indoor Smoking and ARI in Children

**Figure 4:**
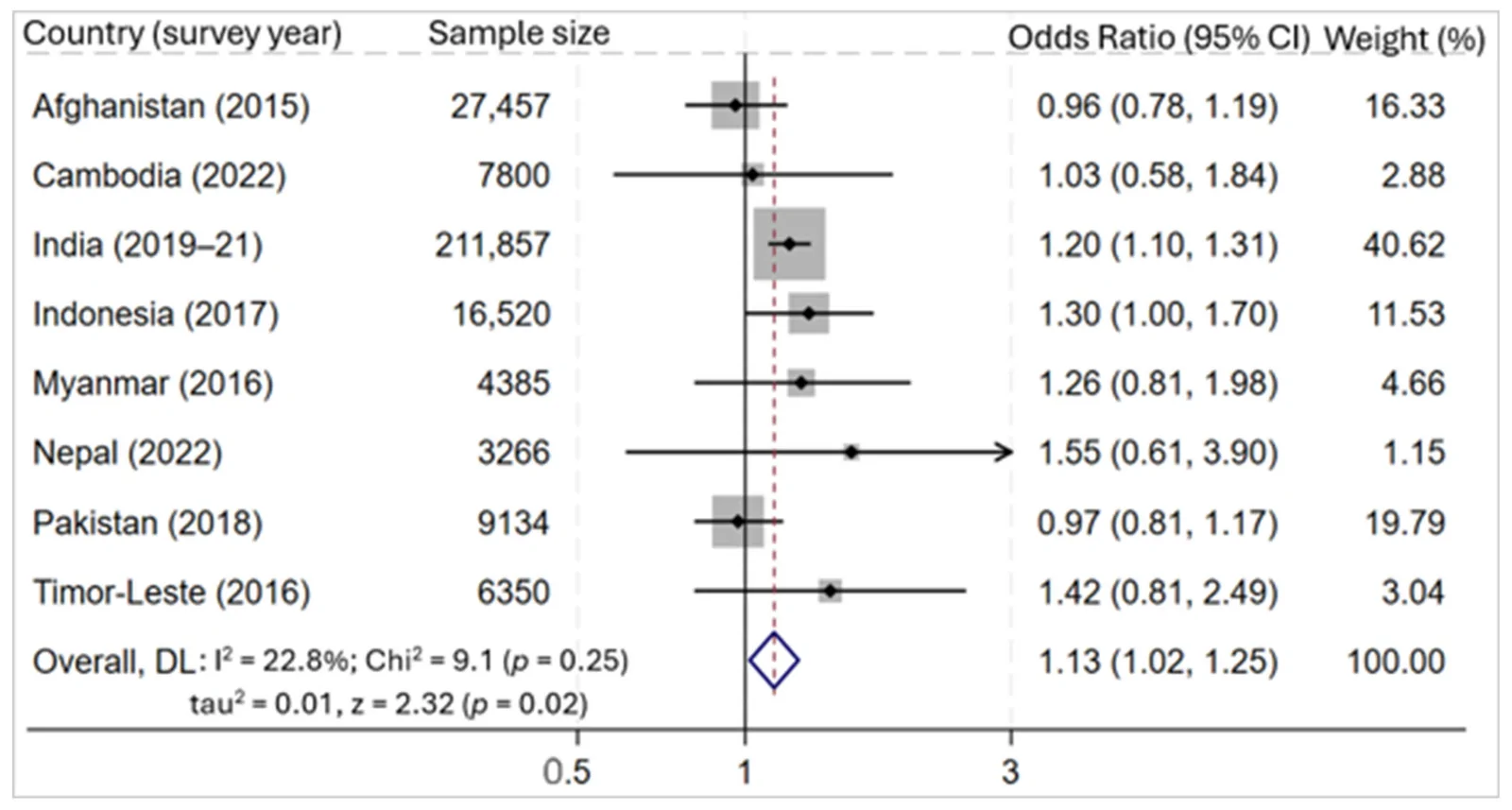
Relationship between any household member daily indoor smoking and odds of ARI in children aged <5 years compared with counterparts living in non-smoking households in the Asia-Pacific countries. The diamond symbols and size of the shaded grey boxes indicate effect size and the contribution of country-specific estimates in the meta-analysis, respectively, and horizontal lines indicate 95% CIs. The centre of the blue diamond denotes the pooled OR, and the corners indicate the 95% confidence intervals. The sample size shown in the figure is unweighted.

Higher odds of ARI associated with living with any household member smoking indoors daily were observed in six of the eight countries, but this result was statistically significant only for India (OR = 1.20, 95% CI: 1.10, 1.31) (Table S5). The pooled estimate of ARI associated with household members’ daily indoor smoking was significant (pOR = 1.13, 95% CI: 1.02, 1.25). The I^2^ value (22.8%) suggests that there is no important heterogeneity between estimates (Figure 4). The narrower 95% prediction interval (0.92, 1.38) shows the effect is more consistent across countries, but since it includes values close to 1, there might be no effect in some settings.

**Figure 5:**
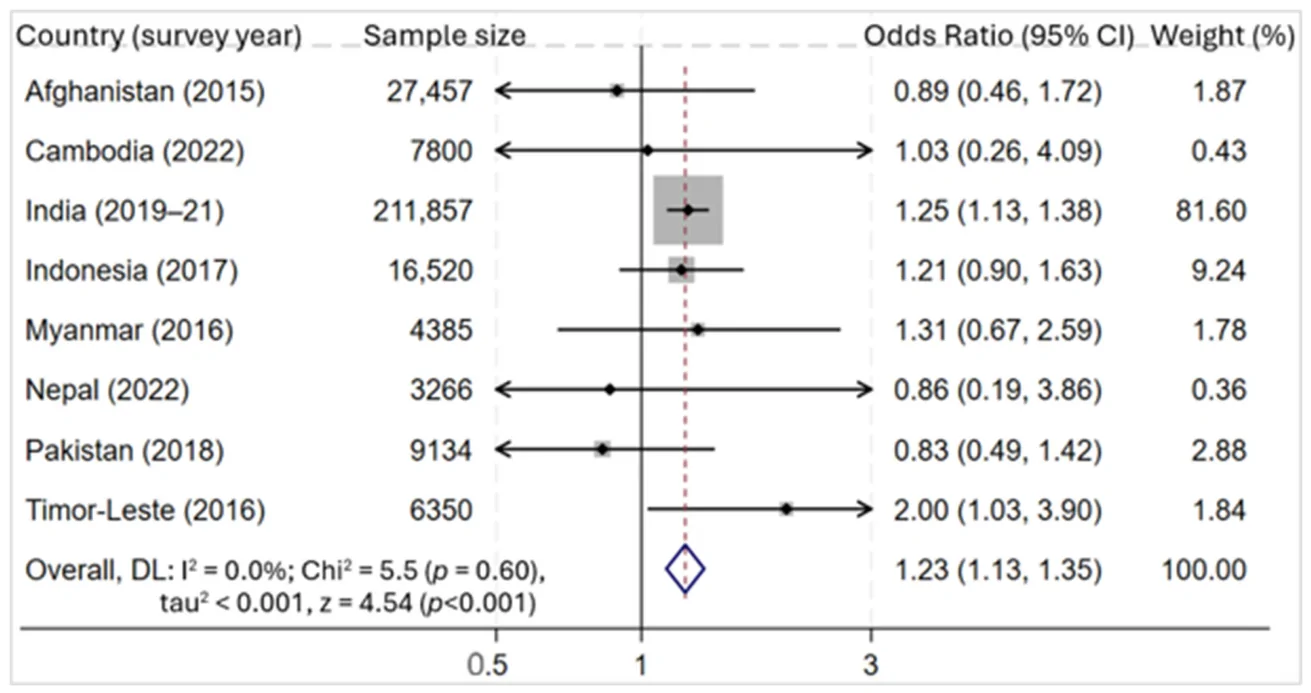
Relationship between any household member smoking indoors occasionally and odds of ARI in children aged <5 years compared with counterparts living in non-smoking households in the Asia-Pacific, analysed from DHS data. The diamond symbols and size of the shaded grey boxes indicate effect size and the contribution of country-specific estimates in the meta-analysis, respectively, and horizontal lines indicate 95% CIs. The centre of the blue diamond denotes the pooled OR, and the corners indicate the 95% confidence intervals. The sample size shown in the figure is unweighted.

The odds of ARI associated with living with any household member smoking indoors occasionally was significant in Timor-Leste (OR = 2.00, 95% CI: 1:03, 3.90) and India (OR = 1.25, 95% CI: 1.13, 1.38) (Table S5). The pooled estimate of ARI associated with household members smoking indoors occasionally was associated with higher odds of ARI (pOR = 1.23, 95% CI: 1.13, 1.35). The large *p*-value for heterogeneity of 0.60 and the I^2^ of 0% indicate that no important heterogeneity between country-specific estimates is detected (Figure 5). Again, the 95% prediction interval for occasional indoor smoking (1.10, 1.38) excluded the null value, indicating a consistently harmful association across countries with less between-country variability.

There was no substantial difference in the estimates obtained from the random- and fixed-effects models. The pooled estimates from fixed-effects model for maternal tobacco use, daily indoor smoking, and occasional indoor smoking were 1.48 (95% CI: 1.31, 1.66), 1.15 (95% CI: 1.07, 1.23), and 1.23 (95% CI: 1.13, 1.35), respectively (see Figures S2–S4).

## 4. Discussion

Effective tobacco control is essential for protecting children’s health and achieving the Sustainable Development Goals (SDGs), particularly those targeting good health and well-being, reduced inequalities, and environmental sustainability [35]. By applying a harmonised analytical approach to nationally representative DHS data from eight Asia-Pacific countries, this study addresses a key gap in the evidence, namely, the lack of comparable, cross-country estimates linking indoor smoking and maternal tobacco use to childhood ARI. Meta-analytic results indicate that children living in households where household members smoke indoors, either daily or occasionally, were more likely to report ARI than those living in smoke-free households. Similarly, maternal tobacco use was associated with higher odds of ARI in children. The pooled estimate for maternal tobacco use and ARI shows considerable heterogeneity (I^2^ = 78.1%), likely reflecting cross-country differences in social norms around female smoking, tobacco control policy implementation, and survey timing relative to national tobacco control initiatives. Additionally, its 95% prediction interval (0.62, 4.51), which crosses the null, indicates that the true effect in a new, unstudied population could range from a modest protective association to a considerably larger harmful association.

These findings broadly align with results from other studies examining child respiratory outcomes in relation to maternal tobacco use and SHS exposure. A study from India found higher odds of ARI among children of smoking mothers compared with non-smoking mothers [19]. Studies from China [4] and Vietnam [36] similarly reported that SHS exposure increased the risk of adverse respiratory outcomes and hospitalisation for lower respiratory infections, respectively, while a study from Hong Kong found that tobacco smoke exposure increased children’s likelihood of developing respiratory health problems [34]. However, these studies were largely confined to single countries or settings, limiting their ability to capture cross-national patterns. By contrast, the present study draws on the most recent nationally representative DHS data across eight Asia-Pacific countries, enabling harmonised, comparable estimates of the association between tobacco smoke exposure and childhood ARI across diverse socioeconomic and cultural contexts. Further, variation in outcome and exposure measurements across studies means this comparison should be treated with caution.

One notable finding of this study is that occasional indoor smoking was also significantly associated with increased odds of ARI. To our knowledge, previous studies have generally treated indoor smoking exposure as a binary variable, without distinguishing between occasional and daily exposure. This finding, that even occasional indoor smoking is associated with higher odds of ARI, adds to growing evidence suggesting that even low levels of SHS exposure may pose respiratory risks to children—there is no safe level of SHS exposure [37], reinforcing the need for collaborative efforts to protect children from environmental tobacco hazards in the settings where they live [38]. Furthermore, the 95% prediction interval for occasional indoor smoking (1.10, 1.38) did not cross the null, suggesting a positive association is likely to hold future studies, although this should still be interpreted with some caution as the pooled estimate was based on a limited number of studies and was predominantly driven by the Indian estimate, which constrains how far it can be generalised to the other included countries.

Children face a greater risk of developing acute and chronic health problems from tobacco smoke exposure, owing to their higher breathing rate, greater lung surface area relative to body size, and immature immune system [39]. Beyond direct exposure, maternal tobacco use may also reduce breastmilk quality [40], increase toxic chemicals in breastmilk [41], and shorten breastfeeding duration [42], potentially limiting children’s access to the early immune protection breastfeeding provides. Maternal tobacco use should therefore be considered a priority public health concern. SHS exposure remains a leading contributor to respiratory tract infections [43], one of the most significant infectious causes of childhood morbidity and mortality [44]. Future studies could examine whether tobacco-related respiratory illness in children is associated with greater care complexity and healthcare use, integrating clinical and structured care data [45].

Implementation of the WHO FCTC recommendations is central to upholding the right to health [46,47]. Our findings are consistent with the WHO FCTC Article 8 guidelines, reinforcing the MPOWER ‘Protect’ component’s call for comprehensive smoke-exposure reduction [48]. Despite progress in some countries, many still face tobacco industry interference and limited tobacco control capacity [49,50], underscoring the need for stronger regulation, monitoring, and evaluation.

Cessation support, quitlines, and pharmacotherapy should be routinely offered but remain limited in many LMICs, including those examined in this study [51]. For example, among the 182 countries that submitted the WHO FCTC reports in the most recent reporting cycle, only 53 reported cessation programmes for girls and young women, and only 71 reported programmes for pregnant women [52]. None of the countries included in this analysis reported such services. These interventions have strong public support [53] and demonstrated benefits in reducing tobacco-related respiratory illness [54] and cancer risk [55]. These findings support developing, strengthening, and implementing tobacco control policies and cessation programmes that protect children from SHS.

Educating parents and communities about the harms of tobacco use by using culturally appropriate approaches via media campaigns and during antenatal care services is essential. This is particularly important, as parents may underestimate the risks SHS exposure poses specifically to their children’s health. Additionally, comprehensive tobacco control strategies that support smoke-free environments, encompassing both supply- and demand-side measures, are also needed to reduce household tobacco exposure across the region. Robust policies that shield tobacco control efforts from tobacco industry interference are crucial. Together, these measures could reduce tobacco-related healthcare costs from childhood respiratory illnesses and avert productivity losses from premature death and disease [56].

### Strengths and Limitations

This is among the few studies to examine the association of maternal tobacco use and SHS exposure with ARI in children across eight countries in the Asia-Pacific. The analysis draws on the most recent nationally representative data, strengthening the relevance of findings to current country contexts. The inclusion of both country-specific estimates and a pooled meta-analysis provides a comprehensive understanding of the exposure-outcome relationship, enhancing the study’s applicability for each country and for all included countries combined.

This study also has several limitations. The nature of cross-sectional design precludes establishing causality. Although the most feasible approach for obtaining nationally representative data in resource limited settings, maternal-reported ARI symptoms may be subject to recall bias and outcome misclassification. Additionally, self-reported maternal smoking and SHS exposure may have been underreported due to social stigma and cultural norms, potentially leading to underestimation of true exposure prevalence. Future research should consider methods such as hair, serum, and urine cotinine levels to objectively measure such variables. The analysis did not account for vaping, which is increasingly common in LMICs [51] and carries similar respiratory risks [57], nor for thirdhand smoke exposure from residual tobacco chemicals in indoor environments [58]. The included surveys were collected in different years (spanning 2015–2022), and the meta-analytic estimates do not account for temporal variation across surveys. Given the small number of included studies and the wide prediction intervals, our findings should be interpreted with caution, as the true effect in a new population or context could differ from our pooled results. Furthermore, the pooled result for occasional indoor smoking relies heavily on data from India, so it may not reflect the situation in the other included countries. Finally, despite adjusting for a range of known confounders, residual confounding may persist because information on factors such as pre-existing child health conditions, breastfeeding status, and ambient air pollution was unavailable.

## 5. Conclusions

This meta-analysis found that maternal tobacco use and indoor smoking, including occasional indoor smoking, are associated with higher odds of ARI in children. Comprehensive tobacco control measures addressing both the supply and demand sides, guided by the WHO FCTC framework, are needed to reduce this burden. Expanding access to cessation support, alongside coordinated smoke-free laws that extend to private settings, could reduce tobacco-related respiratory illness in children and generate economic benefits by averting healthcare costs and premature deaths.

## Figures and Tables

**Table 1:** Weighted children’s ARI status and maternal tobacco use by Asia-Pacific countries, DHS data.

Countries (Survey Year)	Child’s ARI Status		Maternal Tobacco Use Status	
	No: n (%)	Yes: n (%)	No: n (%)	Yes: n (%)
Afghanistan (2015)	26,455 (87.42)	3807 (12.58)	28,947 (95.89)	1240 (4.11)
Cambodia (2022)	7694 (98.74)	98 (1.26)	7646 (98.12)	146 (1.88)
India (2019–2021)	216,031 (97.21)	6198 (2.79)	214,974 (96.74)	7255 (3.26)
Indonesia (2017)	15,860 (95.82)	693 (4.18)	16,223 (98.01)	330 (1.99)
Myanmar (2016)	3967 (96.80)	131 (3.20)	3904 (95.24)	195 (4.76)
Nepal (2022)	4967 (98.55)	73 (1.45)	4783 (94.89)	257 (5.11)
Pakistan (2018)	8445 (86.19)	1353 (13.81)	9113 (93.04)	682 (6.96)
Timor-Leste (2016)	6929 (98.03)	139 (1.97)	6728 (95.18)	340 (4.82)
**Total**	290,349 (95.88)	12,492 (4.12)	292,317 (96.55)	10,446 (3.45)

## Data Availability

The data used in this study (Demographic and Health Survey) are publicly available and can be accessed through formal request at https://dhsprogram.com. The authors do not have permission to share data. Code availability: Code is available upon request from the corresponding author.

## Supplementary Materials

The following supporting information can be downloaded at: https://www.mdpi.com/article/10.3390/healthcare14193221/s1. Table S1. Asia-Pacific countries under DHS program; Table S2. Outcome, exposure, and covariates included; Table S3. Weighted indoor smoking status in eight Asia-Pacific countries, DHS data (2015–2022); Table S4. Weighted child, maternal, and household characteristics by children’s ARI status, DHS data from eight Asia-Pacific countries, DHS data (2015–2022); Table S5. Logistic regression analysis of the association of maternal tobacco use and exposure to SHS with ARI among under-five children living in eight Asia-Pacific countries, DHS data: Figure S1. The flowchart for the selection of children included in the analysis (unweighted frequencies), DHS data (2015–2022); Figure S2. Odds ratio of child ARI associated with maternal tobacco use in the Asia-Pacific region; Figure S3. Relationship between any household member daily indoor smoking and odds of ARI in children aged <5 years in the Asia-Pacific countries; Figure S4. Relationship between any household member smoking indoors occasionally and odds of ARI in children aged <5 years in the Asia-Pacific, analysed from DHS data.
