# Supplementary material for: Maternal and Indoor Smoking Are Associated with Increased Risk of Acute Respiratory Infection in Under-Five Children: A Meta-Analysis of National Survey Data from Eight Asia-Pacific Countries
Source: Healthcare (Basel). 2026 Sep 30;14(19):3221. doi: 10.3390/healthcare14193221 (PMC13664749; doi:10.3390/healthcare14193221)
Supplement: Supplementary file 1 [file healthcare-14-03221-s001.zip › healthcare-4544157-supplementary.pdf]

Maternal and Indoor Smoking Are Associated with Increased Risk of Acute Respiratory Infection in Under-Five Children: A Meta-Analysis of National Survey Data from Eight Asia-Pacific Countries

Daniel Bogale Odo, Yonatan Dinku, Uday Narayan Yadav, Desalegn Markos Shifti, Zelalem Mengesha, Giridhara Rathnaiah Babu, Bhim Prasad Sapkota and Raglan Maddox

Table S1. Asian Pacific countries under DHS program

Countries	Included/ excluded	Reason
Afghanistan	Included	
Bangladesh	Excluded	No data on maternal smoking
Cambodia	Included	
India	Included	
Indonesia	Included	
Lao People's Democratic Republic	Excluded	No standard DHS data available
Maldives	Excluded	Few mothers use tobacco, inadequate observation
Mongolia	Excluded	No DHS data
Myanmar	Included	
Nepal	Included	
Pakistan	Included	
Philippines	Excluded	Few mothers use tobacco, inadequate observation
Sri Lanka	Excluded	Data not in public domain
Thailand	Excluded	Old data (1987)
Timor-Leste	Included	
Vietnam	Excluded	Old data (2005)

Table S2. Outcome, exposure and covariates included

Acute Respiratory Infection (ARI): whether a child had a short, rapid breathing which was chest-related and/or difficult breathing which was chest-related) in the 2 weeks preceding the survey (yes/no): Specifically, symptoms of ARI was defined as a cough accompanied by short, rapid breathing which was chest-related and/or by difficult breathing which was chest-related (h31b = 1 & h31c in 1,3).

This is implemented in STATA as:

- gen ARI= 0
- replace ARI =1 if h31b==1 & (h31c==1 | h31c==3)
- label define ARI 0"No" 1"Yes"
- label var ARI "Child has acute respiratory infection (ARI)"
- label val ARI ARI
- tab ARI

Maternal tobacco use: mother smokes cigarette or uses other form of tobacco such as shisha, cigars, pipe full of tobacco, etc. (yes/no)

Child sex: whether a child is male or female

Child's age: ranging from 0 year to 4 years old

Maternal education: no education, primary, secondary, and higher

Household wealth index: The wealth index is a composite measure of a household's cumulative living standard, calculated by DHS using household's ownership of selected assets, such as televisions and bicycles; materials used for housing construction; and types of water access and sanitation facilities. The wealth index places individual households on a continuous scale of relative wealth. DHS separates all interviewed households into five wealth quintiles: poorest, poorer, middle, rich and richest).

Cooking fuel: Type of cooking fuel presented as electricity, LPG, natural gas, biogas, kerosene, coal, lignite, wood, animal dung, crop residue, grass/shrubs/straw; categorised into clean: electricity, LPG, natural gas, biogas and the reminder into polluting.

Basic handwashing facility: Households that had a fixed place where household members wash their hands, and those who had water plus soap on premises (confirmed by observation) at the time of the interview are labelled as having basic handwashing facility. This approach is used to estimate global status of basic handwashing facility.

Place of residence: Type of place of residence where the household resides as either urban or rural
Environmental tobacco exposure: frequency household members smoke inside the house (never, daily, weekly, monthly, less than once monthly).
Occasional smoking: it is defined as smoking on a less than daily basis, consistent with the "*Guide to DHS Statistics*", other national tobacco surveys and prior studies.

Table S3. Weighted indoor smoking status in eight Asian Pacific countries, DHS data (2015-2022)

Countries	Frequency of indoor smoking (n=302,841)		
	Daily (%)	Occasionally (%)	Did not smoke (%)
Afghanistan	6505 (21.50)	1193 (3.94)	22,563 (74.56)
Cambodia	2241 (28.76)	312 (4.00)	5239 (67.24)
India	61,031 (27.46)	40,499 (18.22)	120,699 (54.31)
Indonesia	9322 (56.32)	3302 (19.95)	3928 (23.73)
Myanmar	1977 (48.24)	414 (10.09)	1708 (41.67)
Nepal	1166 (23.13)	608 (12.07)	3266 (64.80)
Pakistan	3391 (34.61)	254 (2.59)	6152 (62.80)
Timor-Leste	4026 (56.96)	1239 (17.52)	1804 (25.52)
Total	89,661 (29.61)	47,820 (15.79)	165,360 (54.60)

Table S4. Weighted child, maternal and household characteristics by children's ARI status, DHS data from eight Asian Pacific countries, DHS data (2015-2022)

**Variables**		**India**			**Nepal**		
		**Child's ARI status**			**Child's ARI status**		
		no	yes	total	no	yes	total
Maternal smoking status	no	196,922	5575	202,497	3,906	59	3,965
	%	97.25	2.75		98.51	1.49	
	yes	6,728	287	7015	238	2	240
	%	95.91	4.09		99.32	0.68	
	Total	203,650	5862	209,512	4,145	61	4,205
	%	97.2	2.8		98.56	1.44	
Frequency of indoor smoking	daily	55,449	1789	57,238	985	18	1,003
	%	96.87	3.13		98.19	1.81	
	weekly or less	37,094	1239	38,333	515	7	522
	%	96.77	3.23		98.63	1.37	
	no	111,107	2,834	113,941	2,645	35	2,680
	%	97.51	2.49		98.68	1.32	
	Total	203,650	5,862	209,512	4,145	61	4,205
	%	97.2	2.8		98.56	1.44	
Child's sex	male	105,247	3,298	108,545	2,161	33	2,194
	%	96.96	3.04		98.48	1.52	
	female	98,404	2,564	100,967	1,984	27	2,011
	%	97.46	2.54		98.63	1.37	
	Total	203,650	5,862	209,512	4,145	61	4,205

	%	97.2	2.8		98.56	1.44	
Child's current age	0	38,846	1,354	40,200	748	13	762
	%	96.63	3.37		98.26	1.74	
	1	39,364	1,350	40,714	787	10	797
	%	96.69	3.31		98.77	1.23	
	2	40,490	1,129	41,620	889	11	900
	%	97.29	2.71		98.75	1.25	
	3	41,046	1,073	42,119	885	16	901
	%	97.45	2.55		98.23	1.77	
	4	43,904	956	44,859	834	11	845
	%	97.87	2.13		98.75	1.25	
	Total	203,650	5,862	209,512	4,145	61	4,205
	%	97.2	2.8		98.56	1.44	
Maternal highest education level	no education	43,579.2	1,303.5	44,882.8	834.6	10.6	845.2
	%	97.1	2.9		98.75	1.25	
	primary	24,887	871	25,758	1,458	20	1,477
	%	96.62	3.38		98.66	1.34	
	secondary	103,305	2,969	106,275	1,665	28	1,693
	%	97.21	2.79		98.35	1.65	
	higher	31,879	718	32,597	187	2	189
	%	97.8	2.2		98.72	1.28	
	Total	203,650	5,862	209,512	4,145	61	4,205
	%	97.2	2.8		98.56	1.44	
Mothers' age at the time of the survey in 5-year group	15-19	4,875	191	5066	192	6	198
	%	96.23	3.77		97.19	2.81	
	20-24	59,769	1,883	61,652	1,325	16	1,342
	%	96.95	3.05		98.78	1.22	
	25-29	82,676	2,253	84,930	1,448	25	1,473
	%	97.35	2.65		98.28	1.72	
	30-34	38,801	1,068	39,869	773	9	782
	%	97.32	2.68		98.84	1.16	
	35-39	13,840	369	14,209	314	3	318
	%	97.4	2.6		98.93	1.07	
	40-44	3,025	65	3090	70	1	71
	%	97.89	2.11		98.58	1.42	
	45-49	665.392	31.696	697.088	22.249	0.000	22.249
	%	95.45	4.55		100	0	
	Total	203,650	5,862	209,512	4,145	61	4,205
	%	97.2	2.8		98.56	1.44	
Household wealth index	poorest	48,999	1,624	50,623	1,054	19	1,073
	%	96.79	3.21		98.2	1.8	
	poorer	43,454	1,382	44,836	803	11	814
	%	96.92	3.08		98.59	1.41	
	middle	39,716	1,100	40,816	820	13	833

	%	97.3	2.7		98.43	1.57	
	richer	38,144	948	39,092	791	7	798
	%	97.57	2.43		99.11	0.89	
	richest	33,338	808	34,146	677	10	686
	%	97.63	2.37		98.57	1.43	
	Total	203,650	5,862	209,512	4,145	61	4,205
	%	97.2	2.8		98.56	1.44	
Place of residence	urban	55,758	1,303	57,061	2,664	36	2,700
	%	97.72	2.28		98.67	1.33	
	rural	147,892	4,559	152,451	1,480	25	1,505
	%	97.01	2.99		98.34	1.66	
	Total	203,650	5,862	209,512	4,145	61	4,205
	%	97.2	2.8		98.56	1.44	
Main cooking fuel type	Clean fuel	103,134	2,751	105,884	1,553	19	1,572
	%	97.4	2.6		98.8	1.2	
	Polluted fuel	100,517	3,111	103,628	2,591	42	2,633
	%	97	3		98.41	1.59	
	Total	203,650	5,862	209,512	4,145	61	4,205
	%	97.2	2.8		98.56	1.44	
Basic handwashing facility	No	63,704	1,967	65,672	353	9	363
	%	97	3		97.42	2.58	
	Yes	138,309	3,836	142,146	2,879	33	2,912
	%	97.3	2.7		98.85	1.15	
	Total	202,014	5,804	207,817	3,232	43	3,275
	%	97.21	2.79		98.69	1.31	
**Variables**		**Timor-Leste**			**Pakistan**		
		**Child's ARI status**			**Child's ARI status**		
		no	yes	total	no	yes	total
maternal smoking	no	6,548	108	6,655	7,608	1,200	8,808
	%	98.38	1.62		86.38	13.62	
	yes	308	29	337	535	125	660
	%	91.33	8.67		81.1	18.9	
	Total	6,856	137	6,993	8,144	1,325	9,469
	%	98.04	1.96		86.01	13.99	
Indoor smoking	daily	3,906	78	3,985	2,788	467	3,255
	%	98.03	1.97		85.67	14.33	
	weekly or less	1,189	35	1,224	218	27	246
	%	97.13	2.87		88.94	11.06	
	no	1,761	23	1,784	5,140	831	5,971
	%	98.69	1.31		86.08	13.92	
	Total	6,856	137	6,993	8,146	1,325	9,471
	%	98.04	1.96		86.01	13.99	
Child's sex	male	3,541	80	3,621	4,041	674	4,715
	%	97.79	2.21		85.71	14.29	

	female	3,315	57	3,372	4,105	651	4,756
	%	98.32	1.68		86.31	13.69	
	Total	6,856	137	6,993	8,146	1,325	9,471
	%	98.04	1.96		86.01	13.99	
Child's current age	0	1,414	26	1,440	1,576	287	1,862
	%	98.22	1.78		84.61	15.39	
	1	1,409	34	1,443	1,592	324	1,916
	%	97.65	2.35		83.09	16.91	
	2	1,318	31	1,349	1,612	234	1,846
	%	97.7	2.3		87.31	12.69	
	3	1,375	27	1,402	1,632	280	1,912
	%	98.04	1.96		85.34	14.66	
	4	1,340	19	1,359	1,735	200	1,935
	%	98.6	1.4		89.68	10.32	
	Total	6,856	137	6,993	8,146	1,325	9,471
	%	98.04	1.96		86.01	13.99	
Maternal highest education level	no education	1,727.6	30.8	1,758.5	3,995.5	665.0	4,660.5
	%	98.25	1.75		85.73	14.27	
	primary	1,243	26	1,270	1,291	260	1,551
	%	97.92	2.08		83.22	16.78	
	secondary	3,278	59	3,337	1,775	286	2,060
	%	98.22	1.78		86.14	13.86	
	higher	607	20	627	1,085	114	1,199
	%	96.75	3.25		90.49	9.51	
	Total	6,856	137	6,993	8,146	1,325	9,471
	%	98.04	1.96		86.01	13.99	
Mothers' age at the time of the survey in 5-year group	15-19	165	5	170	211	45	256
	%	97.05	2.95		82.4	17.6	
	20-24	1,201	21	1,222	1,578	275	1,853
	%	98.31	1.69		85.15	14.85	
	25-29	2,056	31	2,086	2,575	414	2,990
	%	98.54	1.46		86.14	13.86	
	30-34	1,806	42	1,848	2,166	344	2,510
	%	97.72	2.28		86.29	13.71	
	35-39	860	18	878	1,184	176	1,360
	%	97.98	2.02		87.05	12.95	
	40-44	593	21	614	323	57	379
	%	96.61	3.39		85.07	14.93	
	45-49	174.392	0.000	174.392	109.262	13.135	122.397
	%	100	0		89.27	10.73	
	Total	6,856	137	6,993	8,146	1,325	9,471
	%	98.04	1.96		86.01	13.99	
Household wealth index	poorest	1,380	27	1,407	1,829	330	2,159
	%	98.12	1.88		84.72	15.28	

	poorer	1,403	23	1,426	1,568	312	1,880
	%	98.37	1.63		83.41	16.59	
	middle	1,347	21	1,368	1,704	280	1,984
	%	98.44	1.56		85.9	14.1	
	richer	1,376	31	1,408	1,602	209	1,811
	%	97.77	2.23		88.44	11.56	
	richest	1,350	34	1,384	1,443	194	1,637
	%	97.52	2.48		88.16	11.84	
	Total	6,856	137	6,993	8,146	1,325	9,471
	%	98.04	1.96		86.01	13.99	
Place of residence	urban	1,976	51	2,027	2,613	397	3,010
	%	97.48	2.52		86.83	13.17	
	rural	4,880	86	4,965	5,533	928	6,461
	%	98.27	1.73		85.63	14.37	
	Total	6,856	137	6,993	8,146	1,325	9,471
	%	98.04	1.96		86.01	13.99	
Main cooking fuel type	Clean fuel	645	15	661	3,663	543	4,206
	%	97.69	2.31		87.1	12.9	
	Polluted fuel	6,210	122	6,332	4,483	782	5,265
	%	98.08	1.92		85.14	14.86	
	Total	6,856	137	6,993	8,146	1,325	9,471
	%	98.04	1.96		86.01	13.99	
Basic handwashing facility	No	4,734	100	4,834	2,840	514	3,354
	%	97.93	2.07		84.66	15.34	
	Yes	1,785	35	1,820	4,935	773	5,708
	%	98.09	1.91		86.46	13.54	
	Total	6,519	135	6,654	7,775	1,287	9,063
	%	97.97	2.03		85.8	14.2	

**Variables**		**Cambodia**			**Indonesia**		
		**Child's ARI status**			**Child's ARI status**		
		no	yes	total	no	yes	total
maternal smoking	no	7,498	90	7,588	15,082	663	15,745
	%	98.82	1.18		95.79	4.21	
	yes	139	8	146	307	13	320
	%	94.75	5.25		95.96	4.04	
	Total	7,637	98	7,735	15,388	676	16,064
	%	98.74	1.26		95.79	4.21	
Indoor smoking	daily	2,188	34	2,222	8,646	419	9,065
	%	98.48	1.52		95.38	4.62	
	weekly or less	308	4	312	3,057	133	3,191
	%	98.74	1.26		95.82	4.18	
	no	5,141	60	5,201	3,685	124	3,809
	%	98.85	1.15		96.75	3.25	
	Total	7,637	98	7,735	15,388	676	16,064

	%	98.74	1.26		95.79	4.21	
Child's sex	male	3,871	55	3,927	7,793	363	8,156
	%	98.59	1.41		95.55	4.45	
	female	3,766	42	3,808	7,596	313	7,908
	%	98.89	1.11		96.05	3.95	
	Total	7,637	98	7,735	15,388	676	16,064
	%	98.74	1.26		95.79	4.21	
Child's current age	0	1,608	17	1,625	2,970	96	3,066
	%	98.95	1.05		96.87	3.13	
	1	1,598	32	1,630	3,150	156	3,306
	%	98.05	1.95		95.29	4.71	
	2	1,467	18	1,485	3,017	161	3,178
	%	98.78	1.22		94.93	5.07	
	3	1,512	17	1,529	3,095	128	3,224
	%	98.91	1.09		96.01	3.99	
	4	1,451	14	1,465	3,156	135	3,290
	%	99.05	0.95		95.91	4.09	
	Total	7,637	98	7,735	15,388	676	16,064
	%	98.74	1.26		95.79	4.21	
Maternal highest education level	no education	829.3	15.9	845.2	166.1	9.9	176.0
	%	98.12	1.88		94.39	5.61	
	primary	3,139	46	3,185	3,921	216	4,137
	%	98.56	1.44		94.78	5.22	
	secondary	3,104	35	3,139	8,939	359	9,298
	%	98.89	1.11		96.14	3.86	
	higher	565	1	566	2,362	91	2,453
	%	99.85	0.15		96.28	3.72	
	Total	7,637	98	7,735	15,388	676	16,064
	%	98.74	1.26		95.79	4.21	
Mothers' age at the time of the survey in 5-year group	15-19	205	4	209	342	16	357
	%	98.28	1.72		95.56	4.44	
	20-24	1,316	24	1,340	2,470	121	2,591
	%	98.2	1.8		95.33	4.67	
	25-29	2,136	31	2,168	3,940	178	4,118
	%	98.55	1.45		95.68	4.32	
	30-34	2,077	16	2,093	4,046	195	4,241
	%	99.23	0.77		95.41	4.59	
	35-39	1,364	18	1,382	3,089	110	3,199
	%	98.71	1.29		96.56	3.44	
	40-44	458	4	462	1,231	51	1,281
	%	99.13	0.87		96.05	3.95	
	45-49	80.350	0.441	80.791	270.906	5.591	276.497
	%	99.45	0.55		97.98	2.02	
	Total	7,637	98	7,735	15,388	676	16,064

	%	98.74	1.26		95.79	4.21	
Household wealth index	poorest	1,629	33	1,662	3,048	201	3,248
	%	98	2		93.82	6.18	
	poorer	1,421	19	1,441	3,075	159	3,234
	%	98.65	1.35		95.08	4.92	
	middle	1,416	21	1,437	3,164	111	3,275
	%	98.53	1.47		96.61	3.39	
	richer	1,630	18	1,648	3,122	114	3,236
	%	98.89	1.11		96.49	3.51	
	richest	1,542	5	1,547	2,980	91	3,071
	%	99.65	0.35		97.02	2.98	
	Total	7,637	98	7,735	15,388	676	16,064
	%	98.74	1.26		95.79	4.21	
Place of residence	urban	3,036	14	3,050	7,513	297	7,811
	%	99.55	0.45		96.19	3.81	
	rural	4,601	84	4,685	7,875	379	8,254
	%	98.21	1.79		95.41	4.59	
	Total	7,637	98	7,735	15,388	676	16,064
	%	98.74	1.26		95.79	4.21	
Main cooking fuel type	Clean fuel	3,968	44	4,012	11,880	489	12,369
	%	98.9	1.1		96.04	3.96	
	Polluted fuel	3,669	53	3,722	3,509	187	3,695
	%	98.57	1.43		94.95	5.05	
	Total	7,637	98	7,735	15,388	676	16,064
	%	98.74	1.26		95.79	4.21	
Basic handwashing facility	No	1,153	16	1,169	1,760	105	1,865
	%	98.61	1.39		94.37	5.63	
	Yes	6,410	72	6,482	13,500	569	14,069
	%	98.88	1.12		95.96	4.04	
	Total	7,563	89	7,652	15,261	674	15,935
	%	98.84	1.16		95.77	4.23	
**Variables**		**Afghanistan**			**Myanmar**		
		**Child's ARI status**			**Child's ARI status**		
		no	yes	total	no	yes	total
maternal smoking	no	25,057	3,553	28,610	3,677	117	3,794
	%	87.58	12.42		96.92	3.08	
	yes	1,026	206	1,232	181	9	190
	%	83.29	16.71		95.02	4.98	
	Total	26,084	3,759	29,842	3,858	126	3,984
	%	87.4	12.6		96.83	3.17	
Indoor smoking	daily	5,658	779	6,438	1,851	68	1,919
	%	87.89	12.11		96.45	3.55	
	weekly or less	1,053	126	1,180	384	14	398
	%	89.29	10.71		96.37	3.63	

	no	19,447	2,853	22,300	1,623	44	1,667
	%	87.2	12.8		97.38	2.62	
	Total	26,159	3,759	29,918	3,858	126	3,984
	%	87.44	12.56		96.83	3.17	
Child's sex	male	13,415	1,981	15,395	1,996	76	2,072
	%	87.13	12.87		96.33	3.67	
	female	12,744	1,778	14,522	1,862	50	1,912
	%	87.75	12.25		97.37	2.63	
	Total	26,159	3,759	29,918	3,858	126	3,984
	%	87.44	12.56		96.83	3.17	
Child's current age	0	5,048	691	5,739	767	15	782
	%	87.96	12.04		98.1	1.9	
	1	4,856	784	5,640	788	40	828
	%	86.1	13.9		95.18	4.82	
	2	5,545	961	6,506	725	29	755
	%	85.23	14.77		96.09	3.91	
	3	5,508	698	6,206	821	25	846
	%	88.76	11.24		97	3	
	4	5,202	626	5,827	757	17	773
	%	89.26	10.74		97.85	2.15	
	Total	26,159	3,759	29,918	3,858	126	3,984
	%	87.44	12.56		96.83	3.17	
Maternal highest education level	no education	21,722.8	3,205.4	24,928.2	699.6	22.2	721.8
	%	87.14	12.86		96.92	3.08	
	primary	2,095	315	2,410	1,779	61	1,840
	%	86.95	13.05		96.7	3.3	
	secondary	1,900	201	2,100	1,093	35	1,128
	%	90.45	9.55		96.9	3.1	
	higher	441	38	479	287	8	295
	%	91.99	8.01		97.17	2.83	
	Total	26,159	3,759	29,918	3,858	126	3,984
	%	87.44	12.56		96.83	3.17	
Mothers' age at the time of the survey in 5-year group	15-19	864	100	964	87	5	92
	%	89.62	10.38		95	5	
	20-24	6,874	919	7,792	637	24	662
	%	88.21	11.79		96.34	3.66	
	25-29	7,995	1,243	9,238	1,052	28	1,080
	%	86.55	13.45		97.41	2.59	
	30-34	4,656	751	5,407	955	29	985
	%	86.11	13.89		97.01	2.99	
	35-39	3,761	480	4,242	724	23	747
	%	88.67	11.33		96.96	3.04	
	40-44	1,332	173	1,505	314	13	328
	%	88.49	11.51		95.94	4.06	

	45-49	677.671	92.747	770.418	87.728	3.986	91.714
	%	87.96	12.04		95.65	4.35	
	Total	26,159	3,759	29,918	3,858	126	3,984
	%	87.44	12.56		96.83	3.17	
Household wealth index	poorest	4,788	925	5,713	1,134	46	1,180
	%	83.81	16.19		96.12	3.88	
	poorer	5,351	720	6,072	854	32	886
	%	88.14	11.86		96.44	3.56	
	middle	5,517	805	6,322	658	20	677
	%	87.27	12.73		97.08	2.92	
	richer	5,547	706	6,253	648	18	666
	%	88.71	11.29		97.29	2.71	
	richest	4,956	603	5,559	564	11	575
	%	89.15	10.85		98.07	1.93	
	Total	26,159	3,759	29,918	3,858	126	3,984
	%	87.44	12.56		96.83	3.17	
Place of residence	urban	6,155	829	6,984	868	28	896
	%	88.13	11.87		96.92	3.08	
	rural	20,003	2,930	22,934	2,989	99	3,088
	%	87.22	12.78		96.8	3.2	
	Total	26,159	3,759	29,918	3,858	126	3,984
	%	87.44	12.56		96.83	3.17	
Main cooking fuel type	Clean fuel	8,132	902	9,034	687	18	705
	%	90.02	9.98		97.45	2.55	
	Polluted fuel	18,027	2,857	20,884	3,171	108	3,279
	%	86.32	13.68		96.7	3.3	
	Total	26,159	3,759	29,918	3,858	126	3,984
	%	87.44	12.56		96.83	3.17	
Basic handwashing facility	No	16,914	2,662	19,576	961	43	1,004
	%	86.4	13.6		95.74	4.26	
	Yes	7,070	808	7,878	2,848	82	2,930
	%	89.74	10.26		97.19	2.81	
	Total	23,984	3,470	27,454	3,809	125	3,934
	%	87.36	12.64		96.82	3.18	

Table S5. Logistic regression analysis of the association of maternal tobacco use and exposure to SHS with ARI among under-five children living in eight Asia-Pacific countries, DHS data

	**India** unweighted sample: 211,857				**Pakistan** unweighted sample: 9,134			
	OR	lci	uci	p-value	OR	lci	uci	p-value
Maternal tobacco use status (RG: no)								
yes	1.36	1.16	1.59	0.00	1.44	1.06	1.97	0.02
Child's sex (RG: male)								
female	0.83	0.78	0.89	0.00	0.93	0.79	1.11	0.42
Child's current age (RG: <1)								
1	0.99	0.89	1.10	0.82	1.13	0.87	1.46	0.36
2	0.81	0.73	0.90	0.00	0.80	0.61	1.04	0.10
3	0.74	0.67	0.83	0.00	0.93	0.74	1.19	0.58
4	0.62	0.56	0.70	0.00	0.63	0.48	0.83	0.00
Number of household members	0.99	0.98	1.01	0.27	1.00	0.99	1.02	0.72
Mothers' age at the time of survey (RG: 15 - 19)								
20-24	0.90	0.72	1.12	0.33	0.87	0.51	1.51	0.63
25-29	0.85	0.68	1.06	0.14	0.81	0.50	1.33	0.41
30-34	0.89	0.71	1.12	0.32	0.86	0.51	1.45	0.57
35-39	0.86	0.67	1.11	0.25	0.76	0.44	1.32	0.33
40-44	0.70	0.48	1.03	0.07	0.96	0.49	1.90	0.91
45-49	1.50	0.92	2.43	0.11	0.57	0.23	1.40	0.22
Maternal education status (RG: no formal education)								
primary	1.20	1.06	1.36	0.00	1.38	1.04	1.84	0.03
secondary	1.02	0.92	1.13	0.71	1.16	0.88	1.54	0.29
higher	0.86	0.74	1.00	0.05	0.81	0.54	1.22	0.31
Household wealth quintile (RG: 1st)								
2nd	0.98	0.88	1.09	0.75	1.04	0.76	1.40	0.82
3rd	0.89	0.78	1.01	0.07	0.79	0.55	1.15	0.22
4th	0.85	0.73	0.99	0.04	0.62	0.41	0.93	0.02
5th	0.92	0.77	1.11	0.38	0.69	0.41	1.18	0.18
Main fuel type used for cooking (RG: clean)								
Polluted fuel	0.92	0.84	1.02	0.12	0.96	0.73	1.27	0.79
Presence of basic handwashing facility (RG: no)								
Yes	1.01	0.92	1.10	0.85	1.01	0.79	1.29	0.93
Place of residence (RG: urban								
rural	1.22	1.07	1.39	0.00	0.92	0.70	1.20	0.53
Frequency of indoor smoking (RG: never)								
daily	1.20	1.10	1.31	0.00	0.97	0.81	1.17	0.76
weekly or less	1.25	1.13	1.38	0.00	0.83	0.49	1.42	0.50
	**Nepal** unweighted sample: 3,266				**Timor-Leste** unweighted sample: 6,350			
	OR	lci	uci	p-value	OR	lci	uci	p-value
Maternal tobacco use status (RG: no)								
yes	0.50	0.10	2.48	0.40	5.25	2.92	9.42	0.00
Child's sex (RG: male)								

female	1.20	0.59	2.41	0.61	0.75	0.49	1.15	0.19
Child's current age (RG: <1)								
1	0.51	0.15	1.77	0.29	1.33	0.78	2.26	0.29
2	0.71	0.22	2.25	0.56	1.23	0.65	2.33	0.53
3	0.98	0.35	2.76	0.96	1.17	0.58	2.37	0.66
4	0.61	0.20	1.83	0.38	0.75	0.37	1.53	0.44
Number of household members	0.88	0.74	1.05	0.17	0.99	0.93	1.06	0.84
Mothers' age at the time of survey (RG: 15 - 19)								
20-24	0.44	0.10	1.93	0.27	0.55	0.17	1.76	0.31
25-29	0.80	0.16	3.99	0.78	0.44	0.14	1.34	0.15
30-34	0.47	0.06	3.58	0.46	0.69	0.23	2.12	0.52
35-39	0.53	0.08	3.56	0.51	0.68	0.21	2.26	0.53
40-44	0.84	0.07	9.67	0.89	1.07	0.34	3.33	0.91
Maternal education status (RG: no formal education)								
primary	1.26	0.40	3.93	0.69	1.32	0.68	2.54	0.41
secondary	1.41	0.47	4.29	0.54	1.07	0.62	1.86	0.80
higher	1.46	0.23	9.47	0.69	1.92	0.72	5.09	0.19
Household wealth quintile (RG: 1st)								
2nd	0.91	0.33	2.49	0.85	0.87	0.45	1.69	0.69
3rd	0.58	0.18	1.85	0.36	0.87	0.42	1.78	0.70
4th	0.79	0.25	2.46	0.68	1.14	0.52	2.50	0.74
5th	1.62	0.43	6.09	0.48	1.06	0.33	3.33	0.93
Main fuel type used for cooking (RG: clean)								
Polluted fuel	1.76	0.80	3.90	0.16	1.16	0.65	2.07	0.61
Presence of basic handwashing facility (RG: no)								
Yes	0.45	0.18	1.14	0.09	0.87	0.46	1.67	0.68
Place of residence (RG: urban								
rural	1.01	0.49	2.08	0.98	0.79	0.43	1.43	0.43
Frequency of indoor smoking (RG: never)								
daily	1.55	0.61	3.90	0.35	1.42	0.81	2.49	0.22
weekly or less	0.86	0.19	3.86	0.84	2.00	1.03	3.90	0.04
	**Cambodia** unweighted sample: 7,800				**Indonesia** unweighted sample: 16,520			
	OR	lci	uci	p-value	OR	lci	uci	p-value
Maternal tobacco use status (RG: no)								
yes	3.74	2.00	6.99	0.00	0.85	0.42	1.73	0.66
Child's sex (RG: male)								
female	0.74	0.47	1.17	0.20	0.87	0.73	1.05	0.15
Child's current age (RG: <1)								
1	2.12	1.16	3.86	0.02	1.52	1.11	2.08	0.01
2	1.19	0.63	2.27	0.59	1.62	1.19	2.21	0.00
3	1.00	0.47	2.13	0.99	1.27	0.92	1.76	0.15
4	1.03	0.49	2.15	0.95	1.31	0.94	1.81	0.11
Number of household members	0.98	0.88	1.10	0.76	0.95	0.90	1.00	0.06
Mothers' age at the time of survey (RG: 15 - 19)								
20-24	0.95	0.42	2.15	0.90	1.06	0.53	2.12	0.87
25-29	0.85	0.33	2.24	0.75	1.01	0.51	2.00	0.98
30-34	0.43	0.14	1.31	0.14	1.09	0.54	2.20	0.81

35-39	0.75	0.24	2.36	0.63	0.79	0.38	1.62	0.52
40-44	0.44	0.11	1.77	0.25	0.92	0.43	1.98	0.83
45-49	0.25	0.04	1.58	0.14	0.42	0.14	1.25	0.12
Maternal education status (RG: no formal education)								
primary	0.73	0.36	1.49	0.39	0.93	0.47	1.85	0.84
secondary	0.65	0.28	1.52	0.32	0.78	0.39	1.56	0.47
higher	0.19	0.02	1.56	0.12	0.93	0.44	1.93	0.84
Household wealth quintile (RG: 1st)								
2nd	0.75	0.37	1.49	0.41	0.76	0.57	1.00	0.05
3rd	0.71	0.36	1.39	0.32	0.51	0.37	0.72	0.00
4th	0.59	0.23	1.55	0.29	0.54	0.38	0.77	0.00
5th	0.34	0.10	1.16	0.09	0.46	0.31	0.69	0.00
Main fuel type used for cooking (RG: clean)								
Polluted fuel	0.62	0.33	1.15	0.13	0.88	0.69	1.13	0.32
Presence of basic handwashing facility (RG: no)								
Yes	1.11	0.65	1.90	0.70	0.94	0.71	1.24	0.65
Place of residence (RG: urban								
rural	2.86	1.28	6.42	0.01	0.91	0.73	1.13	0.40
Frequency of indoor smoking (RG: never)								
daily	1.03	0.58	1.84	0.92	1.30	1.00	1.70	0.05
weekly or less	1.03	0.26	4.09	0.97	1.21	0.90	1.63	0.21
	**Afghanistan**<br>unweighted sample: 27,457				**Myanmar**<br>unweighted sample: 4,385			
	OR	lci	uci	p-value	OR	lci	uci	p-value
Maternal tobacco use status (RG: no)								
yes	1.39	1.04	1.85	0.03	1.35	0.72	2.54	0.35
Child's sex (RG: male)								
female	0.92	0.82	1.03	0.16	0.71	0.49	1.03	0.07
Child's current age (RG: <1)								
1	1.21	0.96	1.53	0.11	2.60	1.52	4.44	0.00
2	1.21	0.95	1.56	0.13	2.08	1.15	3.76	0.02
3	0.92	0.71	1.20	0.55	1.57	0.84	2.94	0.16
4	0.86	0.67	1.10	0.24	1.12	0.54	2.29	0.76
Number of household members	0.99	0.97	1.01	0.35	1.03	0.96	1.11	0.40
Mothers' age at the time of survey (RG: 15 - 19)								
20-24	1.20	0.81	1.78	0.37	0.59	0.18	1.95	0.39
25-29	1.45	1.01	2.10	0.05	0.47	0.14	1.50	0.20
30-34	1.49	1.02	2.20	0.04	0.55	0.17	1.75	0.31
35-39	1.13	0.75	1.71	0.56	0.55	0.17	1.78	0.32
40-44	1.21	0.77	1.89	0.41	0.76	0.22	2.63	0.67
45-49	1.16	0.68	2.01	0.58	0.87	0.20	3.79	0.86
Maternal education status (RG: no formal education)								
primary	1.05	0.79	1.40	0.72	1.23	0.64	2.38	0.54
secondary	0.80	0.58	1.09	0.16	1.43	0.66	3.09	0.36
higher	0.72	0.36	1.43	0.35	2.07	0.72	5.92	0.18
Household wealth quintile (RG: 1st)								
2nd	0.76	0.61	0.94	0.01	0.91	0.57	1.45	0.69

3rd	0.86	0.64	1.14	0.30	0.74	0.39	1.39	0.34
4th	0.75	0.54	1.03	0.08	0.58	0.27	1.24	0.16
5th	0.87	0.53	1.44	0.59	0.34	0.11	1.02	0.06
Main fuel type used for cooking (RG: clean)								
Polluted fuel	1.41	1.04	1.92	0.03	0.89	0.33	2.37	0.82
Presence of basic handwashing facility (RG: no)								
Yes	0.83	0.66	1.04	0.11	0.73	0.47	1.15	0.17
Place of residence (RG: urban								
rural	0.81	0.52	1.25	0.34	0.79	0.46	1.38	0.42
Frequency of indoor smoking (RG: never)								
daily	0.96	0.78	1.19	0.73	1.26	0.81	1.98	0.31
weekly or less	0.89	0.46	1.72	0.73	1.31	0.67	2.59	0.43

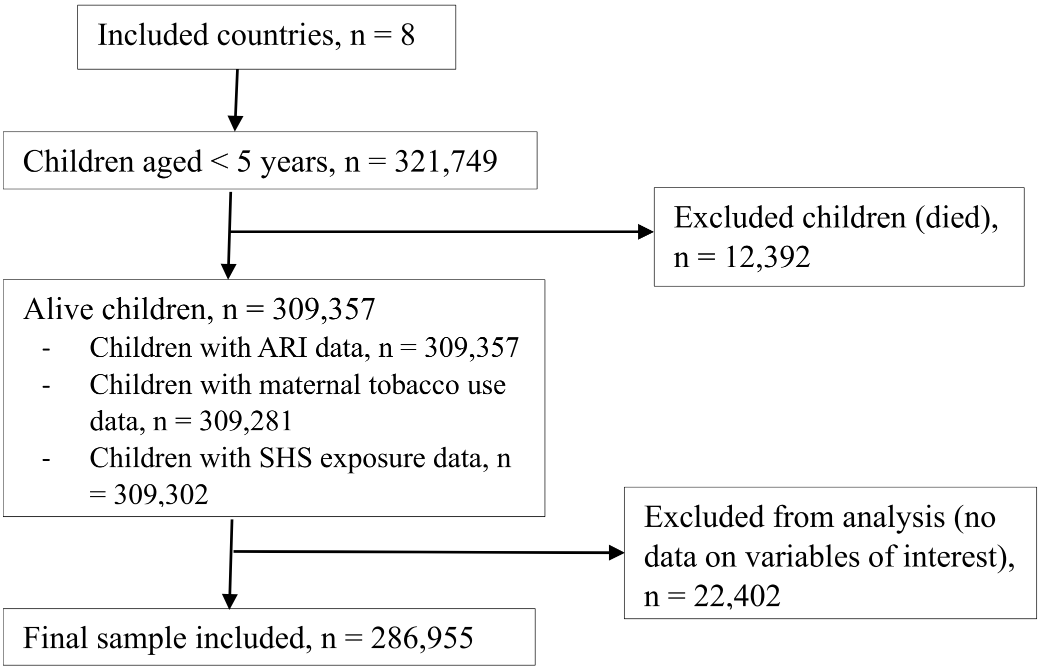

Figure S1. The flowchart for the selection of children included in the analysis (unweighted frequencies), DHS data (2015–2022)

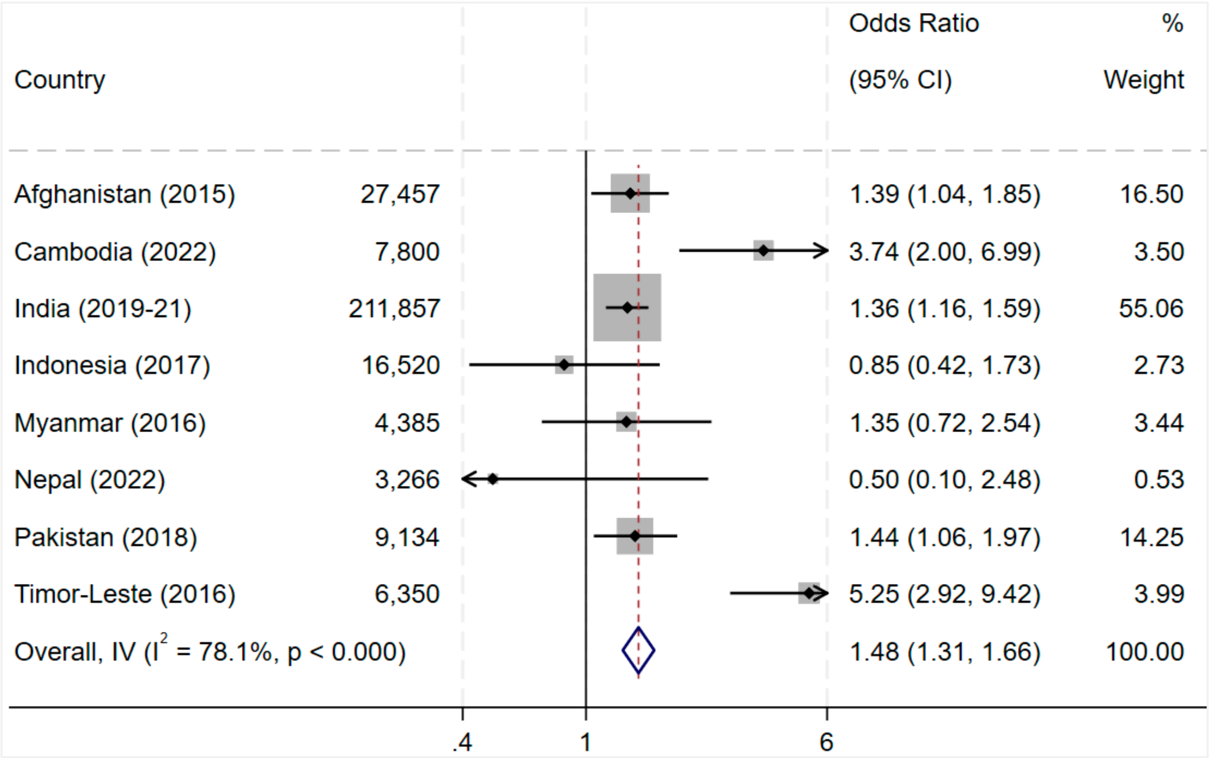

Figure S2. Odds ratio of child ARI associated with maternal tobacco use in the Asia-Pacific region

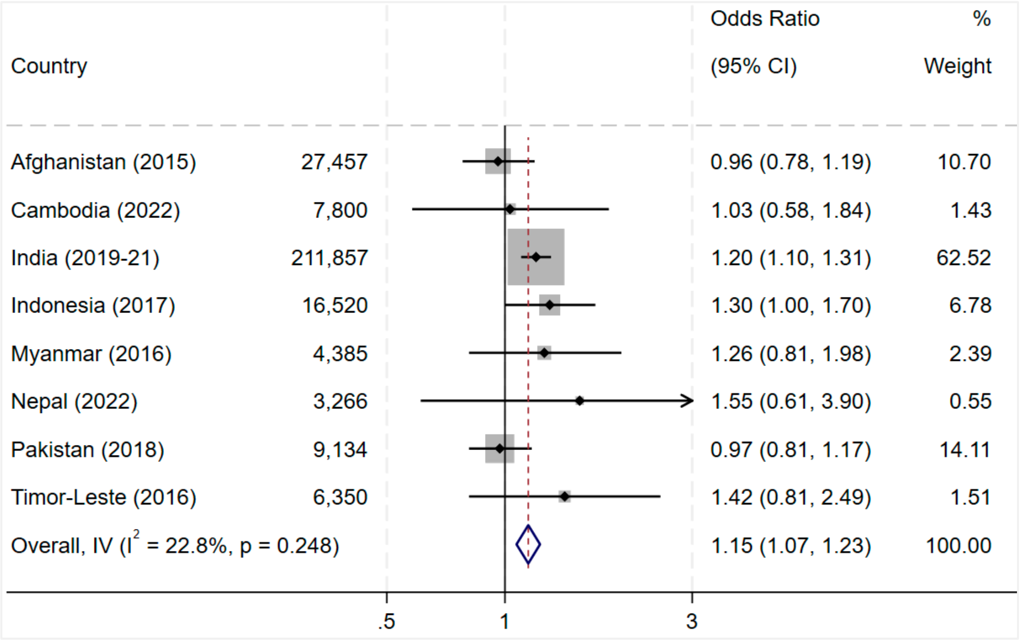

Figure S3. Relationship between any household member daily indoor smoking and odds of ARI in children aged <5 years in the Asia-Pacific countries

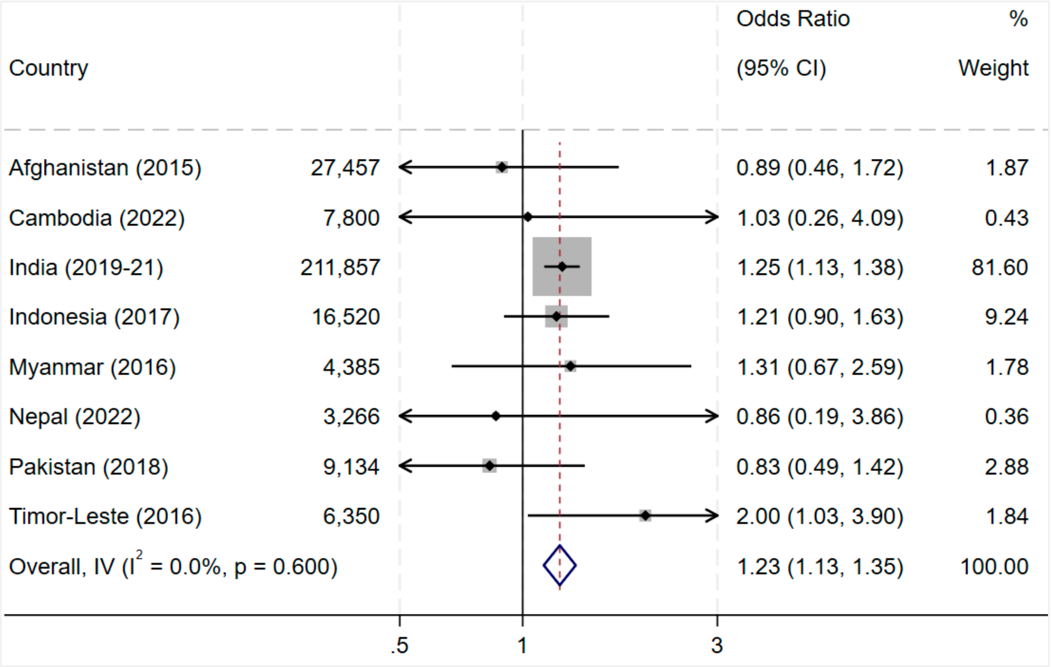

Figure S4. Relationship between any household member occasionally indoor smoking and odds of ARI in children aged <5 years in the Asian Pacific, analysed from DHS data
